# Reporting on covariate adjustment in randomised controlled trials before and after revision of the 2001 CONSORT statement: a literature review

**DOI:** 10.1186/1745-6215-11-59

**Published:** 2010-05-18

**Authors:** Ly-Mee Yu, An-Wen Chan, Sally Hopewell, Jonathan J Deeks, Douglas G Altman

**Affiliations:** 1 Centre for Statistics in Medicine, University of Oxford, Wolfson College Annexe, Linton Road, Oxford, UK; 2 Women's College Research Institute, Department of Medicine, University of Toronto, Canada; 3 Medical Statistics Group/Diagnostic Research Group, Public Health, Epidemiology & Biostatistics, The Public Health Building, The University of Birmingham, Birmingham, UK

## Abstract

**Objectives:**

To evaluate the use and reporting of adjusted analysis in randomised controlled trials (RCTs) and compare the quality of reporting before and after the revision of the CONSORT Statement in 2001.

**Design:**

Comparison of two cross sectional samples of published articles.

**Data Sources:**

Journal articles indexed on PubMed in December 2000 and December 2006.

**Study Selection:**

Parallel group RCTs with a full publication carried out in humans and published in English

**Main outcome measures:**

Proportion of articles reported adjusted analysis; use of adjusted analysis; the reason for adjustment; the method of adjustment and the reporting of adjusted analysis results in the main text and abstract.

**Results:**

In both cohorts, 25% of studies reported adjusted analysis (84/355 in 2000 vs 113/422 in 2006). Compared with articles reporting only unadjusted analyses, articles that reported adjusted analyses were more likely to specify primary outcomes, involve multiple centers, perform stratified randomization, be published in general medical journals, and recruit larger sample sizes. In both years a minority of articles explained why and how covariates were selected for adjustment (20% to 30%). Almost all articles specified the statistical methods used for adjustment (99% in 2000 vs 100% in 2006) but only 5% and 10%, respectively, reported both adjusted and unadjusted results as recommended in the CONSORT guidelines.

**Conclusion:**

There was no evidence of change in the reporting of adjusted analysis results five years after the revision of the CONSORT Statement and only a few articles adhered fully to the CONSORT recommendations.

## Introduction

### Adjusted Analysis in Randomised Controlled Trials

The randomised controlled trial (RCT) is widely accepted as the 'gold standard' design for comparing the effects of health care interventions. Randomisation aims to prevent bias in the allocation of patients to treatment and produce unbiased estimates of treatment effects, but it does not guarantee comparability, particularly in small trials. Adjustment for baseline covariates in the analysis of an RCT is less common than in epidemiological studies. There are four main reasons to consider covariate adjustment methods in RCTs [1-5]: first, to correct for imbalances in baseline prognostic covariates despite randomisation; second, to increase power by modelling the variability in outcome explained by relationships with highly prognostic covariates; third, to obtain treatment effect estimates that would be more closely relevant to individual patients than to an average population; and finally to account for features of study design in the analysis, such as covariates that are used in stratified randomisation. Guidelines suggest that adjusted analysis, including methods of adjustment and choice of covariates, should be pre-specified in the trial protocol [6-8]. In practice, however, adjustment may be done only when baseline imbalance is seen in some covariates [9,10].

### CONSORT Guidance on Adjusted Analysis

The CONSORT Statement, first published in 1996 and revised in 2001, provides recommendations for reporting parallel groups RCTs. It has received considerable support and has been endorsed by many journals and editorial groups worldwide. While briefly mentioned in the 1996 version, the 2001 revision elaborated the recommendations for reporting of adjusted analysis. This includes specification of the rationale for any adjusted analysis, statistical methods used, and clarification of the choice of variables used for adjustment. When reporting results, CONSORT recommends reporting both unadjusted and adjusted analyses, and stating whether the adjusted analysis was planned. However, information on the extent and quality of such practices in published papers is lacking.

In this study, we carried out a systematic review of two cohorts of publications indexed in PubMed to determine the use and reporting of adjusted analysis in RCTs. We also compared the quality of reporting before and after the revision of the CONSORT Statement in 2001.

## Methods

### Study selection

This review included two cohorts: (1) articles published in December 2000 and indexed in PubMed, as previously identified by Chan et al [11,12]; (2) a newly identified cohort of articles indexed in December 2006 in PubMed (as of 22 March 2007). Both cohorts were identified by searching PubMed using the extended version of Phase 1 of the Cochrane Highly Sensitive Search Strategy for trials [13]. The abstracts of the search results for December 2006 were screened by one of the authors (LY). Based on the abstract, all articles that were obviously not trials were excluded. The full text of all remaining articles was fully reviewed (LY) to assess their eligibility.

We included in this review RCTs of parallel group design with a full publication carried out in humans and published in English. Articles published as a letter or brief communication, and articles reporting phase I or pilot studies were excluded. We also excluded studies that did not provide sufficient information on statistical analysis or did not perform any formal comparison between treatment groups.

### Defining adjusted analysis

We identified all trial outcomes that were explicitly reported to have undergone adjusted analysis for comparisons between randomised groups in either the Methods or Results section of the article. We sought mention of the statistical analysis of the treatment effect accounting for covariates or an explicit statement that some results were adjusted. Analyses that used multiple regression methods to identify prognostic variables or risk factors were not defined as adjusted analysis.

### Data extraction

Information on trial characteristics and all outcomes were extracted from the 2006 articles using the same definitions as those in the 2000 cohort [13]. Briefly, the primary outcome reported in the articles was defined if it was explicitly specified in the article, an outcome used in the power calculation, or a main outcome described explicitly in the primary study objectives. Multi-center involvement was defined as data being collected from more than one study site; sample size was defined as the total number of participants randomised in the study.

To maintain independence of observations, we selected one outcome for each trial if more than one outcome underwent adjusted analysis. We selected the outcome according to the following hierarchy: (1) it was a pre-specified primary outcome; (2) the sample size of the trial was based on this outcome; or (3) it had most information on adjusted analysis reported in the article. If more than one outcome was equally reported within an article, then the outcome was chosen at random.

For articles in both cohorts reporting adjusted analysis we assessed the types of analysis reported explicitly in the Methods and Results sections. Articles were classified as reporting unadjusted analysis, adjusted analysis, both, or unspecified/unclear. We also recorded the reason for adjustment, the method of adjustment, and details of the covariates used in the analysis. We assessed whether the unadjusted or adjusted results, such as summary statistics, confidence intervals (CI) or standard error (SE) within group, treatment effect, CI/SE of treatment effect, and the corresponding P-value were reported in the main text and abstract. If results reported in the abstract were not clear, we referred to the main text for type of analysis used.

We also evaluated whether the reporting of adjusted analyses adhered to the 2001 CONSORT guidelines. For the 2006 cohort, we assessed whether articles were published in a CONSORT endorsing journal based on the journals' 'Instruction to Authors' (assessed June 2008). Data regarding trial characteristics were extracted by two reviewers (LY and SH), while outcome and adjusted analysis information were extracted by a single reviewer (LY).

### Data Synthesis and Analysis

Frequency of adjusted analysis was expressed as the proportion of trials that reported using adjusted analysis. Comparisons of trial characteristics and adherence to the 2001 CONSORT Statement between 2000 and 2006 were carried out by Chi-square test for categorical data or Fisher's exact test if expected counts were less than five, and Mann-Whitney test for continuous data. Percentage difference and corresponding precision based on 95% confidence intervals (CI) were calculated to quantify the change in reporting between 2000 and 2006. Similar analyses were used for comparisons of trial characteristics between trials that did or did not report adjusted analysis within each cohort. Data were analyzed using Stata 9 (Stata Corporation, College Station, TX, USA) and a P-value of less than 0.05 was considered to indicate statistical significance.

## Results

### Characteristics of trials

**Figure 1:**
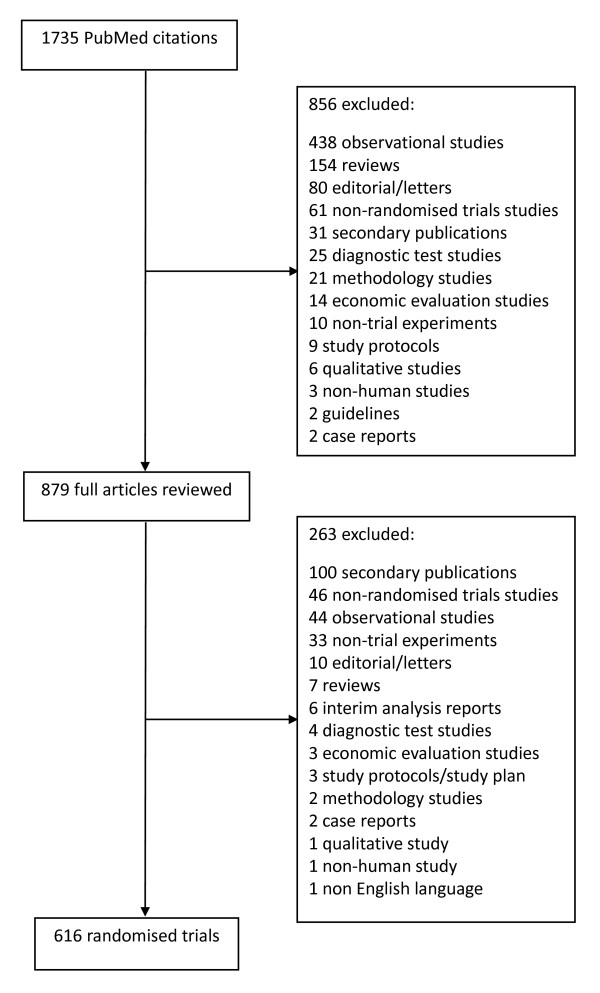
**Flow Chart of December 2006 Articles Eligible for Review**.

**Figure 2:**
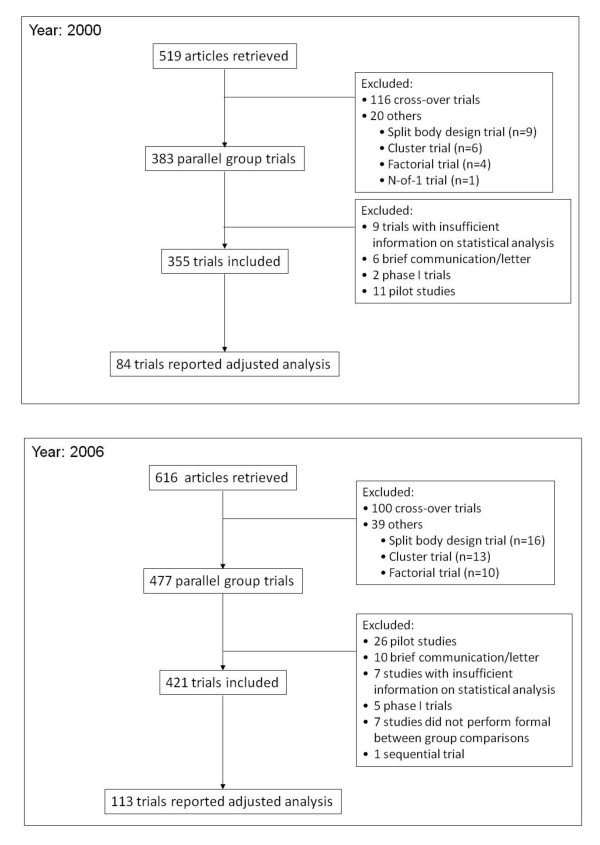
**Flowchart diagram of articles retrieved and included in the review**.

In total, 1735 citations were identified from December 2006 and 616 articles were included. Full details of included and excluded articles are shown in Figure 1. Of the 519 articles retrieved from 326 journals in 2000 and 616 from 316 journals in 2006, 355 and 421 parallel group studies were included in this review, respectively (Figure 2). A significantly lower proportion of articles specified the primary outcome in 2000 than 2006 (51% vs 65%, respectively; P < 0.0001). In both years, most studies were characterized by two study arms (74% for 2000 vs 78% for 2006), a single study centre (both 66%), and publication in specialty journals (both 91%) (Table 1). The average sample size and number of trial outcomes were similar in both years and most reported outcomes were continuous (about 70%). Fewer studies performed stratified randomisation in 2000 than in 2006 (16% vs 20%, respectively; P = 0.1).

**Table 1:** Characteristics of articles of parallel group randomized trials by year of publication

Year of Publication	2000 (n = 355)	2006 (n = 421)	% difference^†^ (95% CI)	P-value
Outcome specification				
Primary	180 (50.7%)	273 (64.9%)	14.1 (7.2 to 21.0)	<0.0001
Unspecified	175 (49.3%)	148 (35.1%)		

Centre involved*				
Multiple centres	119 (34.1%)	138 (33.7%)	-0.4 (-7.2 to 6.3)	0.9
Single centre	230 (65.9%)	272 (66.3%)		

Number of intervention groups	261 (73.5%)	328 (77.9%)		0.2
2	57 (16.1%)	64 (15.2%)		
3	37 (10.4%)	29 (6.9%)		
> 3				

Performed stratified randomisation	56 (15.8%)	85 (20.2%)	4.4 (-1.0 to 9.8)	0.1

Sample size				
< 50	116 (32.7%)	129 (30.6%)		
51 - 150	141 (39.7%)	169 (39.9%)		
151 - 300	49 (13.8%)	52 (12.4%)		
301 - 450	20 (5.6%)	27 (6.6%)		
> 450	29 (8.2%)	44 (10.5%)		
Median (10^th ^to 90^th ^percentile)	91 (27 to 394)	80 (28 to 462)		0.7

Journal type				
General medical	31 (8.7%)	36 (8.6%)	-0.1 (3.8 to -4.2)	0.9
Specialty	324 (91.3%)	385 (91.4%)		

Number of outcomes per trial				
Median (range)	15 (1, 131)	14 (1, 372)		0.2

Type of outcomes	(n = 7132)	(n = 8299)		<0.0001
Continuous	4984 (69.9%)	5705 (68.7%)		
Binary	1961 (27.5%)	2357 (28.4%)		
Time-to-event	47 (0.6%)	128 (1.5%)		
Ordinal	140 (2.0%)	98 (1.2%)		
Categorical	0	11 (0.1%)		

Adjusted analysis	84 (23.7%)	113 (26.8%)	3.1 (-2.9 to 9.3)	0.3

* Unclear: 6 for year 2000 and 11 for year 2006

^† ^Percentage difference = percentage in 2006 - percentage in 2000

Eighty four articles (24%) and 113 articles (27%) in 2000 and 2006, respectively, reported adjusted analyses performed on at least one outcome in the Methods, Results, or both sections.

### Characteristics of trials that did or did not report adjusted analysis

**Figure 3:**
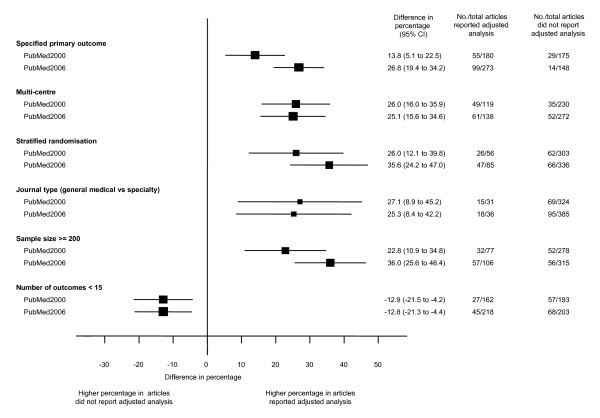
**Comparison of characteristics of articles that did or did not report adjusted analysis for trials published in 2000 and 2006**.

There was a marked difference in the characteristics of studies that did or did not report adjusted analysis in both cohorts. A higher proportion of articles reporting adjusted analysis had specified primary outcomes, involved multiple centers, had performed stratified randomisation, and were published in general journals. Trials with adjusted analysis recruited more participants and had fewer outcomes (Figure 3).

### Consistency of analysis reported between Methods and Results in articles reported adjusted analysis

Among the adjusted analyses articles, 79 and 109 articles had a statistical methods section in 2000 and 2006, respectively. For the outcome selected from each trial, we examined the consistency of the type of analysis reported in the Methods and Results sections. In 2000, 43 out of 79 articles (54%) explicitly specified adjusted analyses were used in the Methods and had subsequently reported them in the Results. Discrepancies between the information in Methods and Results sections were found in 36 articles (46%). For example, two articles had specified adjusted analysis in the Methods but reported only unadjusted results in the Results and 24 (30%) articles did not specify clearly the type of analysis used in the Results section.

In 2006, the consistency of the type of analysis reported in the Method and Results sections increased to 69% (74/109) (P = 0.06; Difference [95% CI] = 13.5% [-0.6% to 27.5%]), while there was a reduction in the proportion of articles that did not specify clearly the type of analysis used in the Results section for the selected outcome (19/109 = 17%) (P = 0.04; Difference [95% CI] = -12.9 (-25.3 to -0.6)). Three articles specified adjusted analysis in the Methods but reported only unadjusted results in the Results. We contacted the authors of the 34 articles with an inconsistency between the Methods and Results section but only three responded.

### Details of adjusted analysis

Details of adjusted analysis are summarized in Table 2. In the 2000 cohort, over 90% of articles had carried out adjusted analysis on the primary outcome. Overall, the majority of articles (80%) did not report the reasons for adjustment or how the covariates were selected for adjustment. Of the 78 articles that specified the covariates, nine (12%) included covariates that were collected after randomisation and 16 (20%) did not specify in the Method section the methods used for adjustment. Fewer than half of the articles included all of the stratification factors used at randomisation in the adjusted analysis. Very few (8% in 2000 and 9% in 2006) specified explicitly whether the adjusted analysis was the primary or secondary analysis.

**Table 2:** Details of adjusted analysis

Year of publication	2000 (n = 84)*	2006 (n = 113)*	% difference (95% CI)	P-value
Performed adjusted analysis on primary outcome^†^	50 (90.9%)	93 (93.9%)	30.0 (-5.9 to 12.0)	0.5

Reason for adjustment				0.4
Imbalance in covariates	9 (10.7%)	12 (10.6%)		
Prognostic covariates	6 (7.1%)	15 (13.3%)		
Both	0	3 (2.6%)		
Other reasons ^‡^	3 (3.6%)	4 (3.5%)		
Not mentioned	66 (78.6%)	79 (69.9%)		

Choice of covariates				0.5
All pre-specified	5 (5.9%)	8 (7.1%)		
All suggested by data	12 (14.3%)	20 (17.7%)		
Combination of pre-specified and post hoc	0	3 (2.6%)		
Not mentioned	67 (79.8%)	82 (72.6%)		

Number of covariates adjusted for ^§^				0.02 ^||^
1	39 (46.4%)	36 (31.8%)		
2	23 (27.4%)	33 (29.2%)		
3-5	14 (16.7%)	25 (22.1%)		
6-9	2 (2.4%)	12 (10.6%)		
Not mentioned	6 (7.1%)	7 (6.2%)		

Covariate used for adjustment				
Outcome assessed at baseline	33/62 (53.2%)	55/81 (67.9%)	14.7 (-1.4 to 30.7)	0.07
Centre/Country	31/49 (63.3%)	25/61 (41.0%)	-22.3 (-40.6 to -39.9)	0.02
Assessed after randomisation	9/78 (11.8%)	9/107 (8.4%)	-3.1 (-12.0 to 5.7)	0.5

All stratification factors were adjusted for	11/25 (44.0%)	20/46 (43.5%)	-0.5 (-24.7 to 23.6)	1.0

Explicitly specified nature of analysis				
Primary analysis	2	5		
Secondary/sensitivity analysis	5	5		

Type of outcomes				0.8
Binary	13 (15.5%)	19 (16.8%)		
Continuous	65 (77.4%)	81 (71.7%)		
Ordinal	1 (1.2%)	3 (2.6%)		
Time-to-event	5 (5.9%)	10 (8.9%)		

Adjusted analysis method used was mentioned for specific outcome in the Method section	62/78 ^¶^(79.5%)	88/109 ^¶ ^(80.7%)	1.2 (-10.4 to 12.9)	0.8

*One adjusted analysis selected per study only

^†^Number of studies that have specified primary outcomes: Year 2000 = 55 and Year 266 = 99

^‡^Year 2000: Clinical relevance (n = 1), significant at 3 weeks after randomisation (n = 1), exploring role of baseline variables (n = 1); Year 2006: Mediated treatment effect on outcome (n = 1), related to compliance/adherence of treatment (n = 2), effect of outcome decline over time (n = 1)

^§^Year 2000: 6 studies did not report number of covariates; Year 2006: 6 studies did not report number of covariates and 1 stated at least 2 covariates

^|| ^Mann-Whitney test

^¶^Did not have statistical method section: Year 2000 (n = 6), Year 2006 (n = 5)

Eighty three articles (99%) in 2000 reported the statistical methods used for adjustment. Since outcomes were predominately continuous, most studies used regression methods (ANCOVA, ANOVA or multiple regressions) for adjustment (Table 3). Binary outcomes and time-to-event data were analysed mainly by logistic regression and Cox regression, respectively. Stratified analyses (e.g. Cochrane-Mantel-Haenszel or Chi-squared analysis) for adjustment were used more often for binary outcomes than other types of outcomes.

**Table 3:** Methods used in adjusted analysis

Year of Publication	2000 (n = 84)	2006 (n = 113)
Continuous data	65 (77.4%)	81 (71.7%)
ANOVA/ANCOVA	*50 (76.9%)*	*56 (69.1%)*
Multiple regression method*	*7 (10.8%)*	*19 (23.5%)*
Stratified analysis	*1 (1.5%)*	*0*
Other^†^	*6 (9.2%)*	*6 (7.4%)*
Not mentioned	*1 (1.5%)*	*0*

Binary data	13 (15.5%)	19 (16.8%)
Logistic regression	*6 (46.1%)*	*11 (57.9%)*
Stratified analysis (Cochrane-Mantel-Haenszel test)	*5 (38.5%)*	*5 (26.3%)*
Other^‡^	*2 (15.4%)*	*3 (15.8%)*

Ordinal data	1 (1.2%)	3 (2.6%)
Stratified analysis (Cochrane-Mantel-Haenszel test)	*1 (100%)*	*1 (33.3%)*
Nonlinear mixed effect model	*0*	*1 (33.3%)*
Ordinal logistic regression	*0*	*1 (33.3%)*

Time to event data	5 (5.9%)	10 (8.9%)
Cox proportional hazard	*5 (100%)*	*9 (90.0%)*
Stratified log rank test	*0*	*1 (10.0%)*


* Including random effect and mixed effect models

^† ^Including GEE, GLM, ANCOVA for rank data, Zellner seemingly unrelated regression, Poisson model, Van Elteren test

^‡ ^Including non-parametric Generalized mixed effect model, GEE, non-parametric ANCOVA

In the 2006 cohort, there was no evidence of change in the reporting of the reason for adjustment (30%) and choice of covariates (27%). More trials in 2006 had adjusted for covariates that were believed to be correlated with the outcomes (13% vs 7%) but only two articles explicitly stated that the covariates selected for adjustment were pre-specified. In addition, more covariates were adjusted for than in 2000, especially outcomes collected at baseline, but fewer multi-centre studies had adjusted for centre effect. Use of statistical methods was similar in both cohorts (Table 3).

### Reporting of adjusted analysis

Table 4 presents the type of results reported in the Results section and abstract. Fifty four articles in 2000 reported any results of adjusted or unadjusted analysis in the Results section. Of these, 80% reported explicitly the type of analysis used to derive the P-values while just under a half reported estimated treatment effects (e.g. odds ratio or difference between means) and the corresponding confidence intervals. Lack of reporting of results, for the selected outcome, in the abstract was more severe. Over 80% of the articles did not report either the treatment effect or the corresponding confidence interval in the abstract. Even P-values were reported in only 31% of the studies.

Overall, there was an increase in reporting any adjusted results in the abstract in 2006 when compared with the 2000 cohort (Table 4). However, in both years a high percentage of articles which used adjustment did not report any adjusted treatment effect. Only 26/50 (52%) in 2000 and 61/93 (66%) in 2006 reported the results of any treatment comparison (i.e. treatment effect estimate, confidence interval, or P-value) in the abstract. Of these, 50% and 61% reported any adjusted results, respectively, but in both years only 30% presented the adjusted treatment effect. Confidence intervals were rarely provided.

**Table 4:** Presentation of results in the Results section and abstract for studies reporting adjusted analysis

Year of Publication	Results Section			Abstract		
	
	2000 (n = 54)	2006 (n = 89)	P-value	2000 (n = 71)*	2006 **(n = 101)**^†^	P-value
Summary statistics for each group			0.7			0.5
Unadjusted only	42 (78%)	70 (80%)		26 (37%)	45 (44%)	
Adjusted only	6 (11%)	12 (14%)		3 (4%)	5 (5%)	
Both	4 (7%)	3 (3%)		0	0	
None/not clear	2 (4%)	3 (3%)		42 (59%)	51 (51%)	

Confidence interval/SE within group			0.2			1.0
Unadjusted only	12 (22%)	11 (13%)		2 (3%)	2 (2%)	
Adjusted only	6 (11%)	10 (11%)		2 (3%)	4 (4%)	
Both	1 (2%)	0		0 (%)	0	
None/not clear	35 (65%)	67 (76%)		67 (94%)	95 (94%)	

Treatment effect			0.4			0.1
Unadjusted only	5 (9%)	5 (6%)		3 (4%)	5 (5%)	
Adjusted only	17 (31%)	35 (39%)		5 (7%)	19 (19%)	
Both	4 (7%)	12 (13%)		1 (2%)	1 (1%)	
None/not clear	28 (52%)	37 (42%)		62 (87%)	76 (75%)	

Confidence interval/SE of treatment effect			0.6			0.4
Unadjusted only	6 (11%)	5 (6%)		2 (3%)	6 (6%)	
Adjusted only	16 (30%)	24 (27%)		3 (4%)	17 (17%)	
Both	4 (7%)	10 (11%)		1 (2%)	1 (1%)	
None/not clear	28 (52%)	49 (56%)		65 (91%)	77 (76%)	

P-value for treatment effect			0.2			0.2
Unadjusted only	9 (17%)	8 (9%)		9 (13%)	13 (13%)	
Adjusted only	27 (50%)	52 (59%)		13 (18%)	30 (30%)	
Both	7 (13%)	17 (19%)		0	2 (2%)	
None/not clear	11 (20%)	11 (13%)		49 (69%)	56 (55%)	

*13 studies did not report the selected outcome in abstract

^† ^2 studies did not have abstract and 10 studies did not report the selected outcome in the abstract

### Adherence to the CONSORT guidelines

With regard to how adjusted analysis should be reported according to the revised CONSORT Statement, there was a slight improvement in some items five years after the revision but the overall adherence is still low (Table 5). Although fewer articles in 2000 reported that stratified randomisation was performed, the proportion that adjusted for any stratification variables was in fact higher than in the 2006 cohort (46% in 2000 vs. 35% in 2006).

**Table 5:** Ad herence to the CONSORT recommendations

Year of publication	2000 (n = 84)	2006 (n = 113)	Relative risk (95% CI)	P-value
Have adjusted for any stratification variables*	26 (46%)	30 (35%)	0.76(0.51, 1.14)	0.2

Have specified rationale for any adjusted analysis	18 (21%)	34 (30%)	1.40(0.85, 2.31)	0.2

Have specified statistical method used for adjusted analysis	83 (99%)	113 (100%)	1.0 (0.97, 1.03)	1.0

Have reported results from adjusted analysis only^2^	18 (21%)	29 (26%)	1.20 (0.71, 2.01)	0.5

Have reported results from both adjusted and unadjusted analysis^†^	4 (5%)	11 (10%)	2.04 (0.67, 6.20)	0.3

* n = 56 for Year 2000 and n = 85 for Year 2006

^†^Results included summary in each group, effect size, and confidence interval

Reporting of both adjusted and unadjusted results was poor. Only four out of 84 articles and 11 out of 113 articles in 2000 and 2006, respectively, reported both results. Of 21 articles (25%) in 2000 that mentioned both adjusted and unadjusted analyses, seven reported only the unadjusted results because the results were similar for both analyses. Similarly, 27 articles had performed both analyses in 2006, of which two reported the adjusted results and five reported the unadjusted results because both results were similar. In addition, four studies in that cohort had reported that the significance of treatment effect was different from unadjusted analysis after adjusting for covariates.

In 2006, 65 of the 113 (57%) articles that reported adjusted analysis were published in CONSORT-endorsing journals. Among these, 23 (35%) specified the rationale for the adjusted analysis performed compared with 11 of the 48 (23%) articles from journals that did not endorse CONSORT. The number of articles which reported both adjusted and unadjusted results was slightly higher in CONSORT endorsing journals compared to non endorsing journals (seven vs four articles, respectively).

## Discussion

Our study provides a comprehensive assessment and comparison of the quality of reporting of adjusted analysis before and after the revision of the CONSORT Statement in 2001. In our review, we found that the characteristics of published reports of parallel group randomised trials indexed in PubMed in 2000 and 2006 were similar, though there was a significant improvement in primary outcome specification in 2006. Only a quarter of randomised trials reported any covariate adjustment analysis. The prevalence of adjusted analysis in our broad cohorts is much lower than the 72% reported in a previous review which was restricted to four high impact general medical journals in 1997 [1] and 64% in a recent review conducted by Austin et al [14]. Another review looked at 34 scientific medical journals in 1998 with a high impact factor and reported 31% of articles had specified adjustment for confounding factors [15]. A further study found similar percentage of adjusted analysis in clinical trials of traumatic brain injury [16]. To our knowledge, these three studies are the only previous such studies addressing this issue. By including journals from all specialties, we believe that the frequency of adjusted analysis in our cohorts is representative of the overall randomised trial literature.

We found that analyses specified in the Methods sections did not necessarily reflect how the results reported in the Results section were obtained. Often the method was either not clearly specified or the results were obtained from different analyses from the specified ones. Readers often trust that the results were derived from analyses specified in the Method section. Our findings have shown that further clarification for reporting results is needed; especially in studies involving adjusted analysis.

Although many authors have discussed how adjusting for baseline covariates in the analysis of RCTs can improve the power of analyses of treatment effect and account for any imbalances in baseline covariates [4,5,17-19], the debate on whether this practice should be carried out remains unresolved. Many recommend that the analysis should be undertaken only if the methods of analysis and choice of covariates are pre-specified in the protocol or statistical analysis plan [1,6-8]. Unfortunately, the rationale for adjustment and choice of covariates were missing in most of the articles we reviewed, although there has been an improvement in the overall reporting of adjusted analysis in trial reports published in 2006 compared to 2000. This lack of pre-specification echoes the findings in the recent review carried out by Chan et al [20]. They found that most trials that mentioned adjusted analysis in either the protocol or article had discrepancies between the two (18/28). Among 18 trials with published adjusted analyses, 12 included covariates that were not pre-specified in the protocol ten of which did not mention any adjusted analysis in the protocol.

Most articles that gave their reason for adjustment or choice of covariates were not in accordance with the guidelines' recommendations [6,7]. Few studies performed and reported the adjusted analysis adequately. For example, where procedures such as stratified randomisation or minimisation methods were used, the analysis without adjustment of stratifying variables could over-estimate the standard error of the treatment effect as well as distort the P-value [21]. Our findings indicate that trials that performed these procedures often did not adjust for stratification/minimisation factors. Furthermore, covariates assessed after randomisation require special attention because their relationship with the study outcome could be confounded by treatment; a different analytical approach is needed [6,7,22,23]. However, we found that some trials included such covariates in the analyses, as has been documented by others [24,25].

Generally, the reporting of adjusted analysis was comparable between the two cohorts we reviewed, which represent trials published before and after the revision of the CONSORT Statement in 2001. Reporting of the main results, such as treatment group summary statistics, treatment effect and confidence intervals, as suggested by CONSORT, were often lacking or unclear in both the Results section and abstract. Such deficiencies could be due to the fact that much more attention has been given to other issues, such as adequacy and transparency of sample size calculation, blinding and randomisation methods, etc, that have already been addressed more often in other systematic reviews [26,27]. Treatment effect estimates from unadjusted and adjusted analyses are not directly comparable because the former gives population-averaged estimates of treatment effect while the latter assesses subject-specific estimates, so it is important that these results are reported clearly so that the treatment effect can be interpreted correctly. This argument is most pertinent in analyses of RCTs with non-continuous outcomes because the treatment effect estimate changes when covariates are included in the analysis [3].

There is little previous evidence about the use and reporting of adjusted analysis in RCTs (19). However, two recent studies reported the impact of selective reporting of adjusted estimates in meta-analyses of observational studies [28,29]. Both studies found that the pooled unadjusted effects differed according to whether studies contributed both adjusted and unadjusted estimates to the meta-analyses or only unadjusted effects. To what extent this lack of clarity in reporting adjusted analyses in RCTs could represent reporting bias that may affect subsequent meta-analyses is unclear. We appreciate that unclear reporting of results does not necessarily reflect poor research conduct, but there is clear evidence suggesting that quality of reporting is associated with bias in the estimation of treatment effect [12,30].

We identified slightly better reporting of key methodological items in CONSORT endorsing as opposed to non CONSORT endorsing journals. However, because there was a time-lag between article publication (December 2006) and when the journal 'Instructions to Authors' were assessed (June 2008) these results should be viewed with some caution. A limitation of this study is that, apart from the trial characteristics for the 2006 cohort, data were extracted by a single reviewer. However, the reviewer revisited the data extraction a few months after the first extraction as a quality assurance procedure. We also used slightly different sampling techniques between the two years. The 2000 cohort included all reports of randomised trials published in December 2000 and indexed in PubMed by July 2002 to account for the lag in PubMed indexing. For pragmatic reasons, the 2006 cohort included those trials indexed in PubMed in December (as of March 2007). This meant that we were able to capture our sample of trials within one search but may have missed a small number of trials which were published in December 2006 but indexed in PubMed after March 2007.

In conclusion, there was no evidence of change in the reporting of adjusted analysis results five years after the revision of CONSORT Statement. Furthermore, overall quality of reporting of adjusted analysis and adherence to CONSORT recommendations remain low. The rationale for covariate adjustment, methods of analysis and choice of covariates for adjustment should be fully reported so that readers can assess whether the adjusted analysis has been adequately carried out and, therefore, should be made transparent in the trial reports. Finally, both unadjusted and adjusted results, which analysis represents the primary analysis, and whether the adjusted analysis was pre-specified in the protocol should also be included in the report.

## Competing interests

We declare that we have no conflict of interest. SH and LMY are funded by NHS. DGA is funded by Cancer Research UK.

## Authors' contributions

L-MY, DGA, JJD contributed to the study design. SH, L-MY and A-WC contributed to the data collection. All authors contributed to the interpretation of results and drafting of the manuscript. L-MY performed the statistical analyses and is the guarantor.
